# Supplementary material for: Are carbon emissions trading and green financial instruments synergistic? -Comprehensive quantitative research based on content analysis
Source: PLoS One. 2024 Mar 7;19(3):e0298601. doi: 10.1371/journal.pone.0298601 (PMC10919601; doi:10.1371/journal.pone.0298601)
Supplement: S1 Data — (ZIP) [file pone.0298601.s001.zip › Data/地级市Z_F工作报告.zip!/╣π╢1⁄2/╒╪╟∞╩╨/└┤╫╘╣1⁄2╓┌║┼í╛┬φ┐╦╩2╛▌═°í┐_2005.docx]

位代表：

　　我代表市人民政府向大会作政府工作报告，请予审议，并请政协各位委员和其他列席人员提出意见。

2004年工作回顾

　　去年，在中共广东省委、省人民政府和中共肇庆市委的正确领导下，在市人民代表大会及其常委会和市政协的监督支持下，市政府坚持以邓小平理论和“三个代表”重要思想为指导，树立和落实科学发展观，深入贯彻中共十六大、十六届三中、四中全会和省委九届四次、五次全会精神，按照市第九次党代会和市委九届二次、三次全会的决策部署，团结奋进，狠抓落实，推进改革，注重效益，全市经济提速增效，社会各项事业全面发展，社会大局稳定有序，精神文明建设和民主法制建设取得新成绩，完成或超额完成市十届人大二次会议确定的各项目标任务，向“三年见成效”目标迈出坚实步伐，为“五年大发展”积蓄了后劲。

　　——国民经济发展加快，产业结构进一步优化

　　国民经济实现近年来最大增幅。积极推进三大经济板块建设，国民经济实现整体提速增效。全市实现本地生产总值548.51亿元(现价)，完成年计划100.2%，比上年增长13.2%，比“十五”计划预期目标高2.2个百分点，是1998年以来的最高增速。以工业为主体的第二产业增幅居三大产业首位。一、二、三产业增加值分别实现151.04亿元、195.51亿元、201.96亿元，分别增长5.7%、18.6%、13.7%。全社会固定资产投资144.71亿元，完成年计划105.1%，比上年增长26.1%，创近10年来新高。外贸出口总额12.4亿美元，完成年计划104.4%，增长12.9%，比预期目标高出近5个百分点。

　　工业经济加速发展。深入推进“工业强市”战略，坚持走新型工业化道路，着力壮大工业规模，加大产业调整力度，电子装备制造业得到省的重点扶持，风华集团进入全省20家装备制造业重点企业行列。企业技术创新力度加大，共申报省重点技术创新项目48个。指导和帮助企业开拓市场，加强管理，挖潜降耗，企业经济效益得到提高。全市规模以上工业企业实现销售收入505.21亿元，增长21.4%；实现税利总额23.59亿元，增长14.4%。全市全部工业增加值172.68亿元，完成年计划101.6%，比上年增长19.3%。工业经济成为推动经济增长的主动力。

　　招商引资再掀热潮。积极参与泛珠三角区域合作，大力实施东引西连战略，招商引资力度进一步加大，招商引资工作责任制得到落实；部分地区生产用电价格下调，撤并整合17个路桥收费站，投资环境不断优化，招商引资再上新台阶。2003“肇庆金秋”经贸洽谈会签约项目履约率达74.2 %，实际投入外资民资54.5亿元，亚洲铝业等一批重大项目即将建成投产。协助省在我市成功举办2004粤台经济技术贸易交流会，我市签约项目24宗，合计投资总额4.55亿美元。成功举办2004“肇庆金秋”经贸洽谈会，签约、奠基和开工项目投资总额达338.5亿元，其中新签投资项目146宗，投资总额205.8亿元。肇庆高新区被省政府确定为广东省吸收外资重点园区和广东省山区吸收外资示范园区并给予政策扶持。高新区实际利用外资和出口总额分别增长223.5%和69.5%，继续保持全市招商引资排头兵地位。按新口径全市新批注册合同外资额5.53亿美元，实际利用外资额4.82亿美元，完成年计划158.7%，比上年增长114.3%。引进外地民资和扶持本地民营企业并重，民营经济发展加快。全市规模以上民营工业企业产值增长28.4%，增幅在各种经济成份中最高。

　　县域经济发展势头良好。认真落实支持发展县域经济的政策措施，山区县以推进工业化、城镇化、农业产业化为核心，重点发展县域工业，大力发展特色经济，积极开展以资源招商、以环境招商、以服务招商，引进建设了一批骨干企业，县域经济呈现多年来少有的良好发展势头。怀集县铜铁金属加工产业和封开县水泥建材产业不断发展壮大，广宁县纸业产业链不断延伸扩展，德庆县通过引入外资、民资改造国有企业加快发展，4个山区县规模以上工业增加值增幅都在20%以上，超过了全市平均增幅。城镇化进程加快，完成了《市域城镇体系规划》和20个中心镇的总体规划修编。

　　农业发展水平得到提高。认真贯彻落实中央［2004］1号文精神，农业的基础地位得到巩固。农业结构调整步伐加快，畜牧水产养殖业稳定发展。高度重视粮食生产，完成粮食播种面积322.26万亩(含大豆)，比上年增加5.91万亩，总产量达到省考核标准。农业产业化取得新发展，新增农业龙头企业7家，新引进发展特色优势农业项目81个，引进资金2.8亿元。农村土地第二轮承包工作顺利推进。在全省率先基本完成林地林权登记换发证工作。完成小型水库除险加固工程58宗。抗旱救灾和预防高致病性禽流感疫情取得明显成效。全市实现农业总产值183.3亿元，比上年增长5.8%。

　　体制改革深入推进。坚持抓改革促发展，多渠道筹集改革资金，市直国有企业改革取得突破性进展，全年实施产权改革的国有企业74家，安置职工7477人，大部分生产性工业、商贸企业已经实施转制改革。组建了国资监管机构。财政体制改革、投融资体制改革、要素市场建设等也取得新进展，体制性障碍逐步消除。

　　旅游等第三产业稳步增长。以千里旅游走廊为重点的旅游资源整合力度加大，与周边地区联手启动无障碍旅游区建设，充分挖掘自驾车游等新亮点，肇庆旅游品牌效应进一步增强，旅游业扭转上年因受非典影响出现的下滑局面，实现了恢复性增长。全市城市接待旅游者人数473.8万人次，比上年增长10.2%；旅游营业总收入35.21亿元，增长16.7%。完成房地产开发投资17.49亿元，比上年增长6.1%。开展了中心城区商业网点发展规划研究。肇庆车城等一批大型服务项目建成开业。交通运输、仓储、邮电通讯业和社会服务业等第三产业持续发展。

　　财税收入实现较大增幅。积极培植税源，抓好增收节支，财税征管得到强化，财税收入实现较快增长，超过GDP增幅。全市地方财政一般预算收入按可比口径为17.18亿元，完成年计划104.8%，比上年增长18.9%，比本地生产总值增幅高5.7个百分点；一般预算支出完成35.86亿元，增长12.5 %。全市国税系统完成税收收入18.2亿元，比上年增长27.1%；地税系统完成税收收入12.8亿元，增长21.2%。土地储备和公开拍卖取得重大突破。

　　——城市规划建设力度加大，交通基础设施建设加快

　　城市规划建设步伐加快。启动了《肇庆市概念性总体规划》和宋城文化景区规划编制以及“山、湖、城、江”城市特色研究，形成了中心城区空间拓展的规划方案。城区道路改造和“美化、绿化、亮化”工程取得阶段性重大进展，端州一、二、三、六路等一批道路扩建改造工程完成，新增绿化面积3万平方米，市容市貌焕然一新。景丰联围达标加固工程涉及的拆迁、征地工作进展顺利，建设步伐加快。

　　新一轮交通基础设施建设全面铺开。通往广州新机场快速干线完成可行性研究。珠三角外环高速公路肇庆段可行性研究报告通过专家评审。广贺高速公路三水至怀集段工程列入国家高速公路主干道规划并正式动工建设。肇庆大桥南引道改造加紧进行。国道321线“文明样板路”创建工程德庆至封开试验路段正式启动建设。县道448线、457线等出省通道改造基本完成。县通镇公路上等级改造和镇通行政村公路硬底化改造完成了年度计划任务。肇庆至南江口文明样板航道建设通过交通部验收。西江大桥扩建工程完成过半。封开开南大桥项目列入广梧高速公路连接线。肇庆新港工程进展顺利。肇庆西江大桥至虎跳门3000吨级江海直达航道整治进入收尾阶段，至两广省界2000吨级航道整治和绥江牛歧船闸改建工程前期准备工作基本就绪。

　　——“十项民心工程”顺利实施，人民生活水平不断提高

　　“十项民心工程”取得阶段性成效。农村费税改革不断深化，降低农业税税率3个百分点，全年减轻农民负担2828万元。加快农村危房改造，解决了近3000户困难群众住房问题。大力推进农村改水、改厕，完成农村饮水工程55宗，解决了17.2万群众饮水难问题。教育扶贫力度加大。法律援助制度进一步完善。农村卫生基础设施不断完善，已有140万人参加农村合作医疗。劳动保障监察工作进一步加强，为3.23万名劳动者追回拖欠的工资押金2932.1万元，共清理拖欠农民工工资3395万元。积极组织旱灾地区生产自救，妥善解决受灾群众特别是困难家庭的生产生活问题。

　　社会保障和就业工作取得新成绩。社会保险扩面征缴力度加大，参加企业养老、失业、基本医疗保险的人数分别增加到25.18万人、26.48万人、28.56万人；共征收社会保险费7.1亿元，比上年增长13%。社会保险市级统筹基本完成。城乡低保政策得到落实，切实做到应保尽保。妥善处理了行政性公司退休人员生活待遇问题。提高劳模荣誉津贴标准，切实解决困难劳模看病难问题。提高了企业离退休人员特别是企业早期退休人员养老待遇。企业退休人员社会化管理服务工作加快推进。五保户供养政策经费纳入财政预算供给。市、县、镇三级公共就业服务体系进一步完善，下岗失业人员小额担保贷款等促进就业和再就业政策得到较好落实。全市城镇新增就业岗位3.67万个，下岗失业人员实现再就业0.97万人，其中“4050”人员0.26万人。城镇登记失业率2.8%。组织培训农民工2.66万人，农村劳动力转移就业40多万人。

　　城乡居民收入稳定增长。全市城镇居民人均可支配收入9261元，比上年增长15.2 %；农村居民人均纯收入4340元，增长4.6 %。全市城乡居民储蓄存款余额293.0 6亿元，增长14.1%。城乡消费畅旺，物价稳中有升。全市社会消费品零售总额193.91亿元， 增长13.3%。居民消费价格指数比上年上升了3.1个百分点。

　　——科教兴市战略全面实施，可持续发展能力不断增强

　　科技创新能力增强。实施省级以上科技计划60项，获省科学技术进步奖6项。省级以上高新技术企业、民营科技企业分别发展到58家、59家。肇庆高新区正在申报国家级高新区。与中山大学建立了全面合作关系，实施了一批科研合作项目。建成省级以上工程技术研究开发中心13家，省级农业科技创新中心1家。高要砚岗和四会东城成为省级技术创新专业镇试点。专利申请量增长41%。“数字肇庆”建设加快推进，被定为国家电子信息产业基地和全省山区信息化示范市。实施了农村科技信息“村村通”工程试点。

　　教育事业蓬勃发展。制定实施了《关于加快建设教育强市的若干意见》。全市第三批101所学校布局调整工作和56所老区山区学校改造工程加快建设，累计扩建校舍建筑面积66万平方米，普及九年义务教育水平不断提高。德庆德城镇成为省级教育强镇。普通高中发展加快，新增高中学位3000多个，高考上线人数再创历史新高。高等教育和职业技术教育上新水平。肇庆学院被批准设立省示范性软件学院。肇庆卫生学校、工商学校、科技学校升格为高等专科学校，肇庆高级技工学校升格为技师学院，职业技术学校在校生增长29.5%，着手筹划建设广东省职业技术教育培训基地。成功举办第二届“教育百万行”活动，社会各界共捐资8911万元。形成了以政府办学为主，公办学校和民办学校共同发展的多元化办学格局。

　　人口工作和生态环境保护得到加强。圆满完成省下达的人口与计划生育工作任务，全市人口出生率、自然增长率比上年分别下降0.42、0.54个千分点。环境综合整治力度加大，创建国家环境保护模范城市工作进展顺利。水环境质量全部达标，饮用水源水质达标率保持100%。工业污染防治进一步加强，1家企业被列为省清洁生产示范企业，7家企业通过省评估验收。市污水处理二厂和高要、四会污水处理厂进入试运行。城区空气污染指数保持良好以上等次。全市完成造林作业面积34.65万亩， 实现了“三年灭荒”目标。创建林业生态县工作取得明显成效，广宁县成为全省首批林业生态县。建成全国首个湿地公园——肇庆星湖湿地公园。完成西江流域水源涵养林建设面积2.96万亩。新增自然保护区8个。

　　——精神文明和民主法制建设上新水平，社会各项事业全面推进

　　精神文明建设取得新成效。“三个代表”重要思想和科学发展观学习实践活动深入开展。未成年人思想道德建设在巩固中深化发展。“千村生态文明建设工程”全面铺开，建成“生态文明村”205个。应邀向民营企业派驻精神文明建设顾问，民营企业精神文明建设呈现新面貌。以“一社区一特色一品牌”为主题的文明社区创建活动取得新成绩。

　　民主法制建设稳步推进。市人大及其常委会的决议得到认真落实。向市人大及其常委会报告工作制度和向市政协通报情况制度进一步完善，全年共向市人大提交各类专项报告14件，组织办理人民代表建议99件，政协提案134件。重视处理人大代表反映的星湖水质等问题。认真听取各民主党派、无党派人士和各人民团体的意见。重大事项社会公示和听证制度落实力度加大。“四五”普法规划深入实施。基层民主进一步扩大，创建了“民主法治示范村”601个。

　　社会大局保持稳定。维护稳定工作机制进一步健全。“严打”整治力度不断加大，“两抢”、“两盗”等恶性犯罪活动得到有效遏制。社会治安防控网络基本形成，各县(市)110、119、112三台合一工程全部完成。“优秀示范小区”、“无案发小区”、“无毒社区”和“无毒村”建设扎实推进，“禁毒03工程”和打击“六合彩”赌博、非法传销等专项行动取得明显成效。坚决打击“法轮功”等邪教组织。社会矛盾的排查、预防、包案、联动、督查机制不断完善，全市群体性事件比上年下降79%。安全生产形势总体平稳。

　　文化名市建设全面启动。制定落实《关于建设文化名市若干意见》，对文化名市建设作出总体部署。着力打造端砚文化品牌，建成端砚展览馆，成功举办“2004·中国端砚文化节”，荣获“中国砚都”称号。文化与旅游进一步融合发展，举办了以“包公文化”为主题的宋文化系列展示活动，与开封、合肥签署了包公文化旅游框架协议，联合湖南永州、广西贺州共建“文化走廊”，完成丽谯楼、梅庵、阅江楼、崇禧塔四大特色文化景区的规划。文物普查、保护和利用工作得到加强，古宋城墙保护开发力度加大。文艺创作繁荣活跃，创作出版了一批以宣传包公、龙母为主题的文艺作品。文化产业不断发展壮大。被省政府评为“实施省人大山区文化建设议案先进集体”。完成文化、广播电视和新闻出版管理体制改革。扫黄打非成效显著，文化市场进一步规范。

　　其他各项事业有新发展。公共卫生体系建设明显加快，突发公共卫生事件应急处理机制进一步完善，疫情信息网络初步形成，成功防控了高致病性禽流感疫情。公共卫生监督执法水平不断提高，顺利通过国家卫生城市复查。卫生系统医德医风建设经验得到监察部和国务院纠风办的肯定和推广。《全民健身计划纲要》深入实施，建成“肇庆星湖山水体育健身圜”，我市荣获“广东省体育贡献奖”，冼东妹为我市夺得首枚奥运金牌。国防动员、兵员征集和民兵预备役工作进一步加强。连续第四届获得全省“双拥模范城”称号。殡葬改革逐步深化。镇村撤并和村务公开扎实推进。授予第三批荣誉市民。与英国波尔顿市建立友好合作关系。经济普查试点工作顺利完成。审计、妇女儿童、民族、宗教、地方志、社会科学、新闻出版、质量监督、气象、防震、人防、档案、老龄、残疾人、社会救助等工作取得新成绩。

　　——政府行政效能进一步提高，转变机关作风效果明显

　　行政管理体制不断创新。大力贯彻《行政许可法》和《全面推进依法行政实施纲要》，废止了74件与法律法规相抵触的规范性文件，开展了第三轮行政审批制度改革，取消了103项行政审批收费项目。行政执法责任制和行政执法监察工作得到强化。新一轮政府机构改革基本完成，制订完善了突发公共事件应急处理机制，政府社会管理和公共服务职能进一步强化。政务公开全面推行。制定实施了市政府《议事决策规则》、《重大事项报告和通报制度》、《信访处理制度》。制定实施了市本级财政资金拨款管理办法，在5个市直部门实行了国库集中支付改革试点。强化了对市直党政机关事业单位经营性物业和政府贷款资金的监督管理，开展了财政支出效绩评价试点。公共资源配置和特定行业市场化运作加快推进。经营性土地公开拍卖、建设工程公开招投标、国有资产公开交易、政府采购等制度得到严格执行。

　　机关作风建设不断加强。开展了以“为民、务实、清廉”为主题的纪律教育学习月活动和两个《条例》学习贯彻活动。公务员培训工作得到加强。成立了市行政效能监察投诉中心。政府服务水平不断提高，建成“一站式”电子政务网络服务平台，实现部分行政许可项目网上查询和并联审批。

　　政府工作目标责任考核制度更加完善。清理各种评比、达标、表彰活动89个。行风评议方法不断完善，开展了行风评议“回头看”活动。信访工作机制逐步健全。整治教育乱收费、纠正医疗购销和医疗服务中的不正之风等专项治理工作取得明显成效，通过了省对我市所有公路基本无“三乱”达标验收。政府廉政工作得到加强，惩治和预防腐败的工作机制不断完善。领导干部任期经济责任审计和述职述廉制度逐步落实。

　　各位代表，我市过去一年取得的成绩，是中共广东省委、省人民政府和中共肇庆市委正确领导的结果，是各级人民代表大会及其常委会和人民政协监督支持的结果，是全市人民团结奋斗、求真务实、开拓进取的结果，也是社会各界关心支持的结果。在此，我代表市人民政府，向全市工人、农民、知识分子、干部职工、外来务工人员、驻肇单位、驻肇部队、武警官兵以及各界人士，致以崇高的敬意！向关心和支持我市建设的外来投资者、港澳台同胞、海外侨胞和国际友人表示衷心的感谢！

　　总结一年来的工作，我们深刻地认识到，要实现市第九次党代会确定的奋斗目标，必须做到“五个坚定不移”：一是坚定不移地以科学发展观为指导，始终把发展作为执政兴市的第一要务。二是坚定不移地走跨越式发展道路，大胆创新发展思路，实施超常规举措。三是坚定不移地实施“工业强市”战略，壮大经济总量，推动经济结构优化升级。四是坚定不移地实施泛珠三角区域合作战略，大力推进东引西连，把招商引资，发展外资、民资、园区经济放在龙头地位。五是坚定不移地实施区域协调发展战略，努力促进中心城区城市新经济、东南部加工制造业经济、山区县域经济协调发展。同时，我们也清醒地认识到，我市经济社会发展仍然存在一些不容忽视的困难和问题。一是经济总量偏小，缺乏龙头企业和大项目拉动，发展速度与全省平均水平仍有差距。二是产业结构不够优化，体制创新力度不够。三是农业产业化水平不高，农民增收依然缓慢。四是由于银根收紧，项目审批难度增大等原因，少数重点项目资金到位率不高。五是就业再就业压力较大，城乡低收入群众生产生活仍然困难。六是科技、教育、文化、卫生等社会事业仍有不少薄弱环节，城市建设管理水平有待提高。七是政府服务意识、行政效能与跨越发展的要求仍有一定差距，工作责任制还没有完全落实到位。这些都必须引起我们高度重视并采取措施认真加以解决。

2005年工作安排

　　今年是我市实现“三年见成效，五年大发展”目标和“十五”规划的重要一年，也是衔接“十一五”规划的关键一年。我们面临着良好的发展机遇，也面临着不少挑战。全球产业结构调整步伐加快，国际资本和产业加速向我国转移；中央继续加强和改善宏观调控，促使经济保持平稳发展态势；“泛珠三角”战略加快推进，省委、省政府对肇庆高新区给予大力支持，更有利于我市东引西连战略的实施；珠三角产业加速向山区转移，有利于我市加快发展。但是，长三角地区以及省内兄弟市发展势头强劲，我市加快发展的竞争压力增大；体制性障碍尚未得到根除，在一定程度上制约了我市经济自主增长活力；经济结构性矛盾仍然较为突出，影响了我市经济发展的速度和质量。因此，我们要扬长避短，趋利避害，进一步增强把握机遇、抢抓机遇的能力，充分做好应对和化解各种困难、问题的准备，按照市委九届四次全会的部署和要求，贯彻“谋全局、促发展、重为民、抓落实”的工作方针，切实加强产业规划发展和园区建设，加强重大项目规划建设，加强农业和农村工作；着力提高招商引资的质量和水平，提高城镇化水平，提高民营经济发展水平，提高旅游和服务业发展水平；努力实现经济社会协调发展，经济增长速度与结构、质量、效益协调发展，三大板块协调发展，城乡协调发展，人与自然协调发展。

　　政府工作的指导思想是：以邓小平理论和“三个代表”重要思想为指导，认真贯彻中共十六届三中、四中全会和中央经济工作会议精神，认真贯彻落实胡锦涛总书记视察广东重要讲话精神和省委九届六次全会精神，以科学发展观统领经济社会发展全局，全面落实市第九次党代会以来市委的各项工作部署，以建设繁荣活力、文明法治、和谐安康肇庆为目标，抢抓发展机遇，深化各项改革，全力招商引资，壮大经济总量，优化经济结构，提高质量效益；优先发展工业，促进产业升级；强化农业基础，推进农村“三化”；加大交通、能源和城市基础设施建设力度，改善投资环境；积极推进科教兴市战略和文化名市建设，促进社会各项事业发展；坚持依法治市，促进民主法制建设，维护社会稳定；坚持以人为本，切实维护和发展人民群众的根本利益；加强政府自身建设，提高执政能力，努力实现“三年见成效”工作目标，为“五年大发展”打下更坚实基础。

　　 经济社会发展的主要预期目标是：生产总值增长14%；其中第一产业增长5.5%，第二产业增长22%，第三产业增长13%。全社会固定资产投资增长30%；实际利用外资增长20%；外贸出口增长10%；地方财政一般预算收入增长15%；社会消费品零售总额增长13%；新增就业岗位3万个，城镇登记失业率控制在3%以内；城镇居民人均可支配收入增长9%，农村居民人均纯收入增长4.5%左右，人口自然增长率控制在7.6‰以内。

　　为了确保今年目标任务完成，必须抓好以下工作：

　　一、调整优化产业结构，加快发展工业经济

　　坚持实施“工业强市”战略，走新型工业化道路，更加突出地把工业放在牵动全局的战略地位，加快壮大工业经济总量，提高工业增长质量和效益。

　　进一步壮大支柱产业。认真抓好产业调整优化，做好产业引导，充分运用中山大学对我市产业选择和发展研究成果，明确产业发展重点，研究落实做大做强支柱产业的政策措施。大力扶持电子信息、生物制药、食品饮料、建材陶瓷、金属加工、林产化工、装备制造、汽车配件等产业实现集群化发展。扶大做强现有骨干企业，大力支持风华集团、星湖公司、蓝带公司等国有重点企业和鼎丰公司、亚洲铝业等投资规模大、产业链条长的外来投资企业上规模、上水平。

　　进一步提高工业经济质量和效益。大力支持现有各种所有制企业增资扩产，加快技术改造步伐，抓好节能降耗，促进产品结构和产业结构优化升级，扩大生产规模，提高经济效益。支持企业增强自主研发能力，加快科技成果转化和产业化步伐，重点抓好风华集团电子装备制造业基地和IC检测设备技改等重点项目建设。做大做强电子信息和生物制药等高技术产业。积极推进林产化工、纺织、食品等传统优势产业的技术改造。打造一批名牌产品。引进和培育技术含量高、发展前景广阔的高新技术企业。继续推进“数字肇庆”建设，以信息化改造传统产业，推动新型工业化进程。

　　进一步深化国有企业改革。坚持以改革促发展，最大限度地盘活存量资产，鼓励和引导外资、民资参与国有企业的改革、改组、改造。加大国有重点工业企业产权改革力度。完善国有资产监督管理体制，加快制定市属国有资产布局调整的总体规划。落实国有资本出资人职责，加快实施国有资本经营预算制度、国有企业经营绩效考核评价制度和国有企业领导人员激励约束制度。重视调动职工参与改革的积极性，切实保护职工合法权益。

　　二、狠抓招商引资，发展壮大非公有制经济

　　不断提高招商引资质量和水平。坚定不移地把招商引资放在“龙头”地位，切实加强对招商引资工作的组织领导，成立市招商引资工作领导小组，完善各级招商工作机构。进一步加大招商引资力度，除高污染、高危行业需要严格控制外，积极引进内外资、大中小项目，着力引进投资规模大、产业链条长的工业项目尤其是高新技术项目。精心组织2005“肇庆金秋”经贸洽谈会等招商活动。进一步创新招商引资手段，提高政府招商实效，积极实施以商引商、中介招商、网络招商、服务招商，促进现有外资企业增资扩产。进一步完善投资环境，健全招商引资服务体系，切实降低企业营商成本。进一步抓好招商引资责任制、招商引资项目、招商引资政策“三落实”，建立引进外资、民资两套考核指标和督查机制，落实激励措施，促进签约项目的动工、建设和投产。

　　突出抓好工业园区建设发展。加强各类工业园区的规划建设管理，充分发挥各类园区作为招商引资主要载体的作用。重点抓好肇庆高新区的开发建设，加强统筹协调，认真落实省政府把肇庆高新区建设成为广东省吸收外资重点园区的要求和各项扶持政策，及时解决高新区建设发展中的问题；高标准、高水平完善高新区整体规划和各项专项规划；用好各级财政专项扶持资金和金融信贷资金，加快园区基础设施和配套设施建设；积极落实与省国资委和省属大企业的合作，争取引入省属国有大型企业以及国内外行业龙头企业参与园区开发建设；加快建设肇庆加工贸易集中监管园区；创造优良的投资环境，制定优惠的投资政策，提供优质的投资服务，增强园区的综合竞争力，真正使肇庆高新区成为我市招商引资的排头兵和新的经济增长极。

　　大力促进民营经济发展。认真落实省、市扶持民营经济发展的政策措施，加强政府服务，强化政府部门指导和服务职能，进一步优化民营经济发展环境。引进外地民资和激活本地民资并重。大力鼓励支持非公有资本进入基础设施、垄断行业、公用事业以及法律法规没有禁止的其他行业和领域。引导和帮助民营企业加快品牌创立、市场开拓、技术创新、体制重构。抓好行业协会的组建工作。加快建立中小企业服务体系，帮助民营企业解决融资难、用地难问题，扶持发展一批成长型的中小企业，培育壮大龙头民营企业和中小企业群体。

　　三、全面推进农村“三化”，发展壮大县域经济

　　努力提高农业产业化水平。认真落实中央［2005］1号文精神，高度重视农业、农村和农民问题，不断提高农业综合生产能力，促进农业增效、农民增收。加大对农业的投入，进一步加强农业基本设施特别是农田基本建设，巩固提高农业基础地位。优化农产品区域布局，做大做强主导产业和名牌产品。落实支持粮食生产政策措施，鼓励种粮大户发展粮食规模经营。加大对农业龙头企业的扶持力度，积极引进区域带动性强的农业龙头项目。引导发展农民专业合作经济组织。促进优势农产品深度开发加工，提高农产品附加值。完善农产品市场信息网，建设农产品流通“绿色通道”，培育壮大农产品专业批发市场。抓好优势农产品种子种苗基地建设。推动农业技术进村入户。完善农产品质量检测体系。确保农村第二轮土地承包工作通过省的验收。探索建立科学、合理的农村土地流转机制。加快农村集体土地所有权登记发证工作。

　　坚持以工业化统领县域经济发展。选准产业发展方向，扶持发展具有县域特色的资源深加工型、劳动密集型和传统优势型产业，积极发展非资源型工业。加大县域工业招商引资力度，积极承接珠三角产业转移扩张项目。以园区为载体促进产业集聚，积极与珠三角发达地区共建产业转移协作园区。加快在建的陶瓷、服装等产业集聚基地建设。着力发展一批特色工业专业镇。

　　提高城镇规划建设水平。认真落实《广东省城镇化发展纲要》，抓好城镇规划管理，进一步完善中心镇规划修编，继续做好镇村撤并工作。大力推广德庆悦城、高要白土小城镇建设试点经验，加快城镇基础设施和公共服务设施建设，提高城镇管理水平。抓好专业镇技术创新试点。继续深化城镇户籍、社保、教育和建设体制改革，营造有利于农村产业和人口向城镇集聚的良好环境。

　　四、大力发展旅游业，加快发展第三产业

　　以改革为动力发展大旅游。加快旅游体制改革，创新旅游管理体制和旅游投资体制。编制完成全市旅游发展规划。整合文化、旅游资源，高标准、高品位建设以星湖风景名胜区为重点的旅游景区景点，完善千里旅游走廊。抓好市场研究和宣传推介，优化旅游客源结构。深入挖掘具有地方特色的人文资源，深度开发自驾车游、生态休闲游、娱乐探险游等旅游精品项目。加大旅游招商力度，增强旅游配套服务功能，大力开发旅游商品。全力支持建设大型综合旅游项目。加强旅游景区景点管理，巩固创建中国优秀旅游城市的成果。

　　以多元化为手段增强出口竞争力。适应国家出口退税政策调整的要求，积极调整优化出口产品结构，促进地方产品出口。实施多元化出口策略，大力开拓新兴出口市场。发展壮大骨干外贸企业和民营外贸企业。整合优化口岸布局，完善电子通关系统，进一步优化通关环境。

　　以市场为导向培育发展新兴第三产业。生活服务业和生产服务业发展并重。加快发展现代服务业，积极承接港澳服务业的扩张转移。完善物流服务体系，大力引进物流龙头企业，加快建设区域物流基地。加强城区商业、餐饮网点规划建设，扶持发展一批连锁经营企业和中高级专业批发市场。推动城区房地产向东拓展。优化消费环境，培育消费热点，促进消费升级。

　　五、加快城市化步伐，加大基础设施建设力度

　　加强城市规划建设管理。成立城市规划委员会。加快编制概念性城市总体规划、城区控制性详细规划和大型综合旅游项目等重点地段的规划设计，积极构建“山、湖、城、江”相和谐的“一江两岸”大城市格局。适时稳步推进行政区域调整，加快东扩南连。推进中心城区城东新区建设和市政公用设施系列工程。重点抓好牌坊东广场建设，人民北路、矶东路和黄塘东路开通，端州七、八路改造等重点市政工程。积极推进大冲广场扩建及连接出口路段改造工程。加强城市管理，提高综合执法水平，依法整治违章建筑、占道经营、乱摆乱卖和“脏、乱、差”现象，改善市容市貌。继续实施中心城区“美化、绿化、亮化”工程，巩固国家卫生城市、国家园林城市的创建成果。

　　全力推进基础设施建设。构筑东引西连现代立体交通网络。加快实施肇庆通广州新机场快速干线工程项目。全面建设广贺高速公路三水至怀集段。完成西江大桥扩建工程。争取尽快上马建设封开开南大桥及其连接线、珠三角外环高速江门—— 肇庆段和二连浩特 ——广州线怀集境内段工程。推进国道321线“文明样板路”创建工作。实现县到镇通三级以上公路，完成镇通村公路硬底化改造800公里。启动西江肇庆大桥至两广省界2000吨航道整治工程。着手研究筹备广茂铁路肇庆段路线改道。配合省、市电力部门动工建设砚都(肇庆)输变电工程，新建高新区旺新变电站和广宁变电站，增容扩建高要东岸变电站。争取肇庆大型能源项目列入国家规划并启动前期工作。抓好城乡水利防灾减灾工程，加快景丰联围达标加固等水利工程建设。

　　加大生态建设和环境保护力度。建设节约型社会，推进清洁生产，发展循环经济。积极开展林业生态县、生态示范区、生态工业园区、绿色学校、绿色社区、绿色住宅小区等生态创建活动。抓好以北岭山景观林为重点的生态公益林和防火林建设。加强自然保护区和野生动植物及湿地的保护管理。加快土地利用规划修编，切实保护土地资源，抓好土地开发管理和耕地易地开发，确保实现年度耕地占补平衡。贯彻落实省《珠江三角洲环境保护规划》，编制完成《肇庆市环境规划》，继续实施西江和星湖综合整治工程，建设德庆污水处理厂 ，启动广宁、鼎湖污水处理厂工程，力争全市城镇生活污水和生活垃圾无害化处理率都达到50%，严格监管工业污染源，整顿违法排污企业，实现创建国家环保模范城市目标。

　　六、依法治税理财，完善现代市场体系

　　大力培育地方税源。充分发挥好财政政策、资金的引导和调控作用，大力扶持对财政收入增长贡献大的产业，进一步培植税源。支持财税部门严格依法征税，加强税务稽查，坚决打击各种偷逃骗税行为，切实做到应收尽收。强化非税收入管理。健全“收支两条线”制度，把带有垄断性质的行政事业单位经营服务性收费纳入“收支两条线”管理范围。全面清理行政事业单位物业特别是经营性资产，统一收取和安排使用经营收益。加强国有资本经营收益征收管理，逐步建立规范的国资收益管理体系。加快推进公共资源配置和特定行业经营市场化运作，按照“先易后难，分步实施”的原则，上半年重点抓好河砂开采权、采矿权和探矿权、机动车号牌、市政公用设施广告经营权等项目的市场公开操作，下半年全面推开。建立规范的土地储备运营机制，加大土地储备力度，提高土地资源收益，规范土地收益使用的审批程序。

　　强化财政预算约束，严格预算执行。预算安排做到“有保有压”，在保证正常运作的基础上，优先解决重点支出，压缩一般性支出。完善和落实财政收支管理制度。继续推进公共财政管理框架建设，全面实行部门预算管理，加快市级国库集中支付改革。扩大政府采购领域和规模。完善财政支出有效监控机制，进一步扩大财政支出项目绩效评价试点。加强政府投资项目和政府性工程的财务监督审计，落实政府性贷款借用还的各项监管制度。实行市直机关公务员统一发放岗位津贴和统一机关、事业单位公用经费标准。

　　积极拓宽融资渠道。促进银企合作，建立中小企业信用担保体系。推动企业改制上市。争取重点项目进入国家、省计划，增强融资建设能力。抓好农村信用社综合改革，提高抗风险能力。

　　推进现代市场体系建设。落实建设工程招投标、经营性土地使用权公开出让、产权交易公开拍卖等制度，培育发展有形建筑、土地、产权等要素市场。提高要素市场信息化、网络化水平。深化粮食流通体制改革。推进企业和个人信用体系建设。发展各类社会中介组织。继续整顿和规范市场秩序，严厉打击制售假冒伪劣产品、侵犯知识产权等违法行为。

　　七、积极实施科教强市战略，扎实推进文化名市建设

　　推进科技强市建设。编制科技中长期发展规划。加快高新技术产业园区和基地建设，积极做好肇庆高新区申报国家级高新区工作。充分运用与中山大学全面合作的平台，促进产学研结合，推动科技攻关合作。继续推进科技创新体系建设，抓好企业工程技术研究开发中心、农业科技创新中心、中小企业和省市专业镇试点技术创新平台建设。加快建设中国院士广东省肇庆活动之家和各县(市)区生产力促进中心。重视标准化工作。建立防震减灾应急指挥系统和完善地震群测群防网络。继续推进信息化，加强市信息中心网络建设，加快实施农村科技信息“村村通”工程。

　　推进教育强市建设。优先发展教育，加大基础教育投入，加强教师队伍建设，巩固提高普及九年义务教育。完成省第三批中小学布局调整试点学校和省第三批老区山区破危学校改造任务，实施中小学校标准化建设，推进“两高普九”和“教育强镇”试点。抓好普通高中扩建扩招，推进示范性高中建设，争取肇庆中学通过国家级示范性高中评估验收。提高高等学校办学水平，积极支持肇庆学院通过教育部本科教学水平评估。加快肇庆医学高等专科学校和肇庆技师学院新校区规划建设，积极筹建广东省职业技术教育培训基地。鼓励和扶持社会力量办学。

　　推进人才强市建设。做好“十一五”人才规划。打造区域性人才市场，建立人才引进快速通道，大力引进高层次、复合型、高技能的紧缺人才。注重培养专业人才、专业队伍。健全人才评价、选拔和激励机制。支持风华集团和星湖科技公司提高博士后工作站建设水平。

　　推进文化名市建设。加强城乡文化基础设施建设，规划筹建市科技中心、博物馆、图书馆、群众艺术馆。继续深入挖掘宋文化和包公文化、端砚文化、龙母文化，支持各县(市)区打造地方特色文化，进一步拓展文博旅游，积极引资开发宋城文化景区和宋街，加快建设丽谯楼、梅庵、阅江楼、崇禧塔四大特色文化文物景区。全力办好2005国家历史文化名城年会，扩大文化交流合作。拓展文化产业招商，扶持民营文化企业，发展壮大文化产业。继续实施“文化艺术上山下乡”和“农村电影放映2131工程”。推进广播电视“村村通”网络升级。成立文化市场综合执法机构，加强文化市场管理。

　　促进社会各项事业全面发展。以疾病预防控制体系和医疗应急救治体系建设为重点，加快公共卫生体系建设。优化配置卫生资源。建设市疾控中心。调整农村中心卫生院布局，加强农村医疗队伍建设，改造薄弱卫生院，巩固完善农村医疗保健网络。落实市、县财政配套资金，力争农村合作医疗覆盖率提高到60%以上。规范医疗机构药品招标采购管理。加快发展中医药事业。加强公共卫生监督执法管理，提高公共卫生水平。做好食品安全检测工作。加强人口与计划生育工作，建立健全“依法管理、村(居)民自治、优质服务、政策推动、综合治理”的计生工作新机制，继续推进农村计生工作重心向村下移，落实层级目标管理责任制，确保完成人口与计划生育工作目标。发展妇女儿童事业，切实保障妇女儿童合法权益。努力改善全民健身和体育后备人才培训的环境，大力推进全民健身运动，发展优势竞技体育项目。深入开展“双拥”活动。巩固和深化殡葬改革。发展老龄、残疾人事业。增强国防意识，加强民兵预备役和人防建设。全面落实民族政策和《宗教事务条例》。精心组织经济普查。做好外事、侨务、气象、档案、地方志等工作。

　　八、坚持以人为本，切实为民谋利

　　做好就业和再就业工作。坚持城乡并重的就业方针，强化就业再就业工作目标责任制考核。落实扶持就业再就业政策措施，加大就业岗位的开发力度，实施“零就业”家庭就业援助制度和农村贫困户就业扶贫行动。加快劳动力市场建设，完善公共就业服务体系，健全城乡一体的劳动力市场信息网络。加大失业人员培训力度，提高失业人员就业竞争能力和创业能力。加强农民工培训，提高劳务输出组织化程度。强化劳动保障监察和劳动关系协调工作，设立劳动保障维权服务中心和劳动保障信息中心，完善劳动保障突发事件预警机制和处置机制。规范企业工资支付行为，督促企业严格执行最低工资制度和按时足额发放职工工资。

　　健全社会保障网络。完善社会保险市级统筹，逐步建立多层次的社会保障体系。狠抓社会保险扩面征缴，重点提高非公有制企业社会保险参保率。进一步完善养老、医疗、失业、工伤和生育等保险制度，大力推行农民工工伤保险。强化社保基金监管，提高基金支付能力。建立企业年金制度，推进多层次养老保险体系建设，积极探索农村养老保险方式。实施补充医疗保险制度。加强对医疗保险定点机构和定点零售药店管理。严格执行低保政策，切实解决农村五保户和城镇特困户的生活困难。大力推进企业退休人员社会化管理服务工作。

　　继续推进“十项民心工程”。建立健全城乡低收入困难群众社会求助体系。继续清理整顿涉农收费，全面免征农业税，进一步减轻农民负担。重视解决征地拆迁中存在的突出问题，实行征地补偿费存款制度。加快农村特困户的危房改造和农村改水、改厕工程。切实解决城镇特困户的住房困难。创新教育扶贫工作机制，切实解决城乡贫困家庭学生上学难问题。进一步完善法律援助制度。认真解决水库移民安置的历史遗留问题。做好救灾救济工作，发展慈善事业。切实维护外来工的合法权益。完善城市生活无着、流浪讨乞人员救助管理制度。

　　九、提高文明法治水平，建设和谐安康肇庆

　　加强精神文明建设。贯彻《公民道德建设实施纲要》，积极开展“爱国、守法、诚信、知礼”现代公民教育活动。积极探索群众性精神文明创建活动新内容、新形式、新机制，倡导推行科学文明健康的生活方式，弘扬新时期肇庆人精神。加快构建“学习型城市”。加强未成年人思想道德建设和高校思想政治工作。开展“文明诚信企业”创建活动。继续实施“千村生态文明工程”。确保通过省文明城市复查，努力争创全国文明城市。

　　推进民主法制建设。全面推进依法治市工作，建立健全执法责任制，造就公正执法、文明法治的舆论和环境。深入开展法制教育，完成“四五”普法规划，提高全民法制意识和法律素养。完善重大事项社会公示和听证制度。抓好村委会、居委会换届选举。深入开展“民主法治示范村”创建活动，推进村务公开。加强城镇社区建设和社区自治。创新厂务公开方式，加强企业民主管理。推进民营企业工会组织建设。

　　维护社会大局稳定。建立完善群体性事件的预警机制、排查调处机制和应急处置机制，积极预防和妥善处理群体性事件。坚持打防结合，坚决打击各种严重刑事犯罪、黑恶势力和“黄赌毒”、“六合彩”、非法传销等违法犯罪活动。坚持不懈与法轮功等邪教组织作斗争。深入推进社会治安防控体系和基层治安管理网络建设，开展和谐平安社区建设，加强城乡结合部、城市出租屋和重点部位的治安整治。做好信访工作，积极化解人民内部矛盾。健全抢险救援和市区110社会联动机制。落实安全生产责任制，加大安全生产执法力度，消除各种安全隐患，确保生产安全。

　　十、建设为民、务实、清廉政府，进一步提高行政效能

　　自觉接受监督。认真落实人大及其常委会的决议、决定，及时向人大及其常委会报告工作。大力支持人民政协履行政治协商、民主监督、参政议政职能。认真听取民主党派、工商联、无党派人士和人民团体的意见。办好人大议案、人民代表建议和政协提案。加强审计监督。推进政务公开，接受社会监督。

　　严格依法行政。贯彻落实国务院《全面推进依法行政实施纲要》，严格按照法定权限和程序行使权力、履行职责，提高政府行政效能。深入贯彻《行政许可法》，深化行政审批制度改革，简化和规范行政许可程序，强化对行政许可的监督。实施行政执法评议考核制和行政执法过错责任追究制。

　　创新政府行政管理体制。进一步转变政府职能，加快推进政企、政事、政资分开。改进管理经济和社会的方式方法，提高政府领导经济工作、保障公共安全和处置突发事件的能力。抓紧制定政府投资项目管理办法，改革竞争性领域项目审批制度，实行企业投资项目核准制和登记备案制。积极简政放权，除法律、法规明确规定必须由市本级行使的经济管理权限外，其余一律下放到县(市)区。抓好“十一五”规划编制工作，科学谋划“十一五”时期的发展。

　　切实转变工作作风。在政府机关党组织中积极开展保持共产党员先进性教育活动，提高党员干部政治思想素质，做到有理想、有责任、有能力、形象好。加强公务员队伍建设，完善培训、考核、任用机制，提高公务员队伍整体素质。增强服务意识，提高办事效率。严格控制行政事业单位人员编制。抓好机关作风建设，加强调查研究，做到察实情、办实事、求实效，切实帮助解决人民群众生产、生活中的困难和问题。严格执行年度工作考核目标责任制，确保经济发展的各项措施落到实处。不断改进政府服务，加快电子政府建设，充分发挥“一站式”电子政务网络和政府门户网站作用，提高行政效能。

　　加强廉政建设。进一步落实廉政建设责任制。坚持标本兼治、综合治理，建立完善教育、制度、监督并重的预防和惩治腐败体系。全面推行行政首长述职述廉制度和任期经济责任审计制度，推行行政问责制。坚决纠正损害群众利益的不正之风。严肃查处违纪违法案件。

　　各位代表！年初召开的市委九届四次全会，明确了今年和今后一段时期工作的指导思想、目标任务和措施要求，我们要切实抓好各项工作的落实，牢固树立和落实科学发展观，增强大局观念和使命意识，齐心协力，为民务实，开拓创新，为实现市第九次党代会确定的目标任务，建设繁荣活力、文明法治、和谐安康肇庆，实现肇庆跨越发展而努力奋斗!
